# Concentration of Bioactive Phenolic Compounds in Olive Mill Wastewater by Direct Contact Membrane Distillation

**DOI:** 10.3390/molecules26061808

**Published:** 2021-03-23

**Authors:** Rosa Tundis, Carmela Conidi, Monica R. Loizzo, Vincenzo Sicari, Rosa Romeo, Alfredo Cassano

**Affiliations:** 1 Department of Pharmacy, Health and Nutritional Sciences, University of Calabria, 87036 Rende, CS, Italy; rosa.tundis@unical.it (R.T.); monica_rosa.loizzo@unical.it (M.R.L.); 2 Institute on Membrane Technology, ITM-CNR, 87036 Rende, CS, Italy; c.conidi@itm.cnr.it; 3 Department of Agricultural Science, Mediterranean University of Reggio Calabria, 89123 Reggio Calabria, Italy; vincenzo.sicari@unirc.it (V.S.); rosa.romeo@unirc.it (R.R.)

**Keywords:** vegetation waters, membrane contactors, concentration, chemical analysis, antioxidant activity, diabetes, obesity

## Abstract

Olive mill wastewater (OMW), generated as a by-product of olive oil production, is considered one of the most polluting effluents produced by the agro-food industry, due to its high concentration of organic matter and nutrients. However, OMW is rich in several polyphenols, representing compounds with remarkable biological properties. This study aimed to analyze the chemical profile as well as the antioxidant and anti-obesity properties of concentrated fractions obtained from microfiltered OMW treated by direct contact membrane distillation (DCMD). Ultra-high performance liquid chromatography (UHPLC) analyses were applied to quantify some phenols selected as phytochemical markers. Moreover, α-Amylase, α-glucosidase, and lipase inhibitory activity were investigated together with the antioxidant activity by means of assays, namely β-carotene bleaching, 2,2′-azino-bis(3-ethylbenzothiazoline-6-sulphonic) acid (ABTS) diammonium salts, 2,2-diphenyl-1-picrylhydrazyl (DPPH) assay, and Ferric Reducing Activity Power (FRAP) tests. MD retentate—which has content of about five times greater of hydroxytyrosol and verbascoside and about 7 times greater of oleuropein than the feed—was more active as an antioxidant in all applied assays. Of interest is the result obtained in the DPPH test (an inhibitory concentration 50% (IC_50_) of 9.8 μg/mL in comparison to the feed (IC_50_ of 97.2 μg/mL)) and in the ABTS assay (an IC_50_ of 0.4 μg/mL in comparison to the feed (IC_50_ of 1.2 μg/mL)).

## 1. Introduction

Olive oil production is one of the most widespread industrial activities in several countries of the Mediterranean Basin, including European countries (e.g., Italy, Greece, Spain, and Portugal), and Northern Africa countries (e.g., Tunisia, Algeria, Libya, Egypt, and Morocco).

A medium-sized olive oil mill generally produces several cubic meters of wastewater (daily) from the extraction process—wastewater resulting from the olive washing process as well as from olive oil washing procedures. This has considerable environmental impacts due to the production of the large amount of contaminant wastewater and the consumption of water. Wastewater treatment for use in various applications may contribute to sustainable water consumption and conservation of ecosystems. Olive mill wastewater (OMW) is a very rich source of phenolic compounds [1]. Its content is variable, depending on olive varieties, growing techniques, harvesting period, and especially the technology used for oil extraction. The typical value of total phenolic content in OMW is in the range of 1.6–10.7 g of gallic acid equivalent (GAE)/L [2]. The biological properties of these molecules have been widely investigated, and revealed a large spectrum of interesting activities, including antioxidant, anti-inflammatory, anti-proliferative, and hypoglycemic effects [3,4,5,6]. The most abundant phenolic species in OMW are gallic acid and hydroxytyrosol, often found as esters of elenolic acid [7].

There has been increasing interest in recent decades on the recovery of bioactive molecules from OMW, in view of the accepted use of these natural compounds (e.g., as nutraceuticals, and as additives in cosmetics and foodstuffs) [8]. However, these by-products present a complex physicochemical composition and the recovery of target compounds from these sources require several steps (i.e., coagulation and precipitation of impurities, adsorption on resins) based on the use of chemicals, solvents, and high temperatures, which account for a significant part of the total production costs.

The utilization of membrane technologies for separating, purifying, and concentrating bioactive phenolic compounds from OMW is a topic that has been largely investigated in recent years as an alternative to the use of liquid–liquid extraction and chromatographic separations. In particular, the use of pressure-driven membrane operations in a sequential combination with membranes of different molecular weight cut-offs, has been proposed as a valid approach to overcome the wide range of molecular weights of phenolic compounds (which make difficult their recovery with high purities) [9,10].

Among membrane processes, there has been increasing interest in direct contact membrane distillation (DCMD) due to its advantages, including the possibility to operate at atmospheric pressure and at a temperature lower than the normal boiling point of the feed solutions, reduced fouling and concentration polarization compared to other membrane processes, high retention of solutes, possibility of treatment of highly viscous solutions, and low energy consumption. Furthermore, this process is not limited by high osmotic pressure and allows to reach concentration levels similar to those obtained in thermal evaporation [11]. Typical applications have been reported in the treatment of hypersaline waters [12] and radioactive wastewaters [13], concentration of dye solutions [14], and fruit juices [15,16]. In this process a water vapor transfer is promoted through a macroporous hydrophobic membrane under a vapor pressure gradient generated by a temperature difference applied between the two sides of the membrane [17]. The hydrophobic nature of the membrane prevents liquid from entering its pores, promoting the formation of liquid/vapor interfaces at the entrances of the membrane pores. The water transport from the feed to the permeate side can be summarized in three steps: (1) evaporation of the water from the hot feed/liquid vapor interface; (2) vapor transport through the membrane’s pores; (3) condensation of permeated water on the cold permeate liquid/interface side [18].

Following our previous study [19], in which OMW was treated in a sequential design through a combination of microfiltration (MF), nanofiltration (NF), and reverse osmosis (RO) to produce polyphenol-enriched fractions, the present work was undertaken in order to produce a concentrated fraction of phenolic compounds through DCMD of microfiltered OMW. The concentrated fraction was investigated for its chemical profile and bioactivity in order to assess its potential use as antioxidant and anti-obesity formulation.

## 2. Results and Discussion

### 2.1. Analyses of Permeate Fluxes

**Figure 1:**
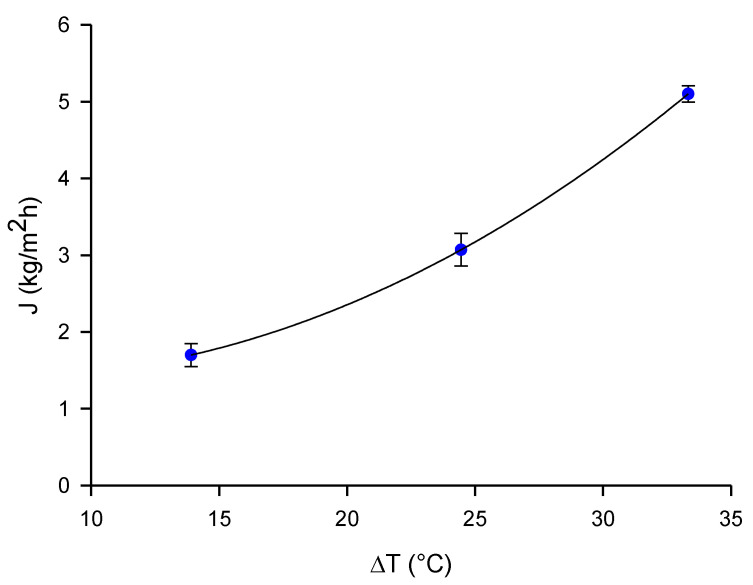
Permeate flux as a function of thermal gradient. Feed, distilled water; Q_feed_, 150 L/h; Q_p_, 80 L/h; T_p_, 10.2 °C.

Preliminary DCMD tests with distilled water were performed to evaluate the permeate flux achievable in the absence of fouling phenomena. Figure 1 shows the variation of the permeate flux with the feed temperature maintaining constant; the permeate temperature at 10.2 °C. An exponential increase of the permeate flux with feed temperature, following an Arrhenius equation, was observed. The average permeate flux values ranged from 1.7 to 5.1 kg/m^2^h.

**Figure 2:**
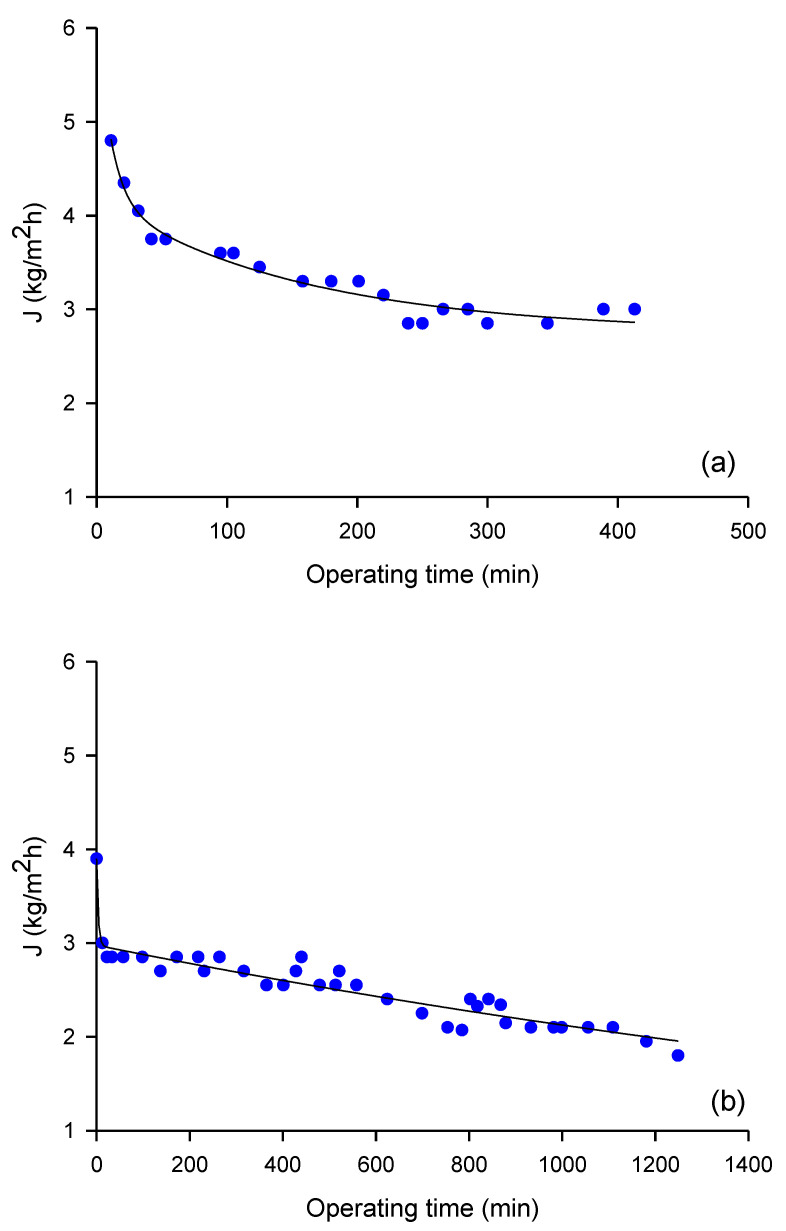
Direct contact membrane distillation (DCMD) of microfiltered Olive mill wastewater (OMW). Time course of evaporation flux up to a weight reduction factor of: (**a**) 3.57 and (**b**) 5.71. Feed, microfiltered OMW; permeate, distilled water; Q_feed_, 150 L/h; Q_p_, 80 L/h; T_feed_, 40.1 °C; T_p_, 10.1 °C.

The membrane productivity measured in DCMD experiments performed with microfiltered OMW under a thermal gradient of 30 °C is illustrated in Figure 2. In particular, Figure 2a shows the time course of the permeate flux measured in DCMD experiments, performed under the selected operating conditions up to a weight reduction factor (WRF) of 3.57. The evaporation flux decreased from 4.8 to 3.0 kg/m^2^h after 6.8 h of microfiltered OMW processing, which means a decrease of 37.5%. In Figure 2b the evaporation flux measured in the same operating conditions, up to a WRF of 5.71, is shown; in this case the evaporation flux decreased from 3.9 to 1.8 kg/m^2^h after 20.8 h of processing, with a decrease of 54% in comparison to the initial value. The decrease of evaporation flux observed in both cases can be attributed to the increase of the feed concentration, polarization concentration effects, and membrane fouling phenomenon [20].

The obtained results are similar to those reported by El-Abbassi et al. [21] in the treatment of crude OMW by osmotic membrane distillation (OMD), with a polytetrafluoroethylene (PTFE) membrane having a pore size of 0.2 μm. The authors reported a decrease in flux from 3.9 to 1.3 L/m^2^h after 30 h of crude OMW processing by OMD, corresponding to a decrease of 67%. The lower decrease in flux observed in our experiments can be attributed to the pretreatment of OMW by MF, which reduces the effect of concentration polarization through the removal of suspended solids from crude OMW. Similarly, Carnevale et al. [22] reported that the permeate flux reduction in the DCMD of OMW pretreated by filtration with a multi-layer tissue did not exceed the percentage of 25% in prolonged tests with capillary polypropylene (PP) membranes of 0.2 μm in pore size. In particular, permeate fluxes decreased from about 7.1 to 5.4 kg/m^2^h after about 20 h under a thermal gradient of 35 °C (T_feed_, 50 °C; T_p_, 15 °C). The lower fluxes obtained in time were attributed to the increase of concentration, which implies a lower water vapor pressure and a more significant effect of the concentration and temperature polarization, as well as membrane fouling. Gryta [23] reported that the intensity of fouling in the concentration of wastewaters containing proteins by MD can be limited by the pre-treatment of feed and a selection of proper operating conditions in MD.

Permeate fluxes obtained in this study resulted much higher than those reported in previous studies, pertaining to the concentration of NF permeate and NF retentate from microfiltered OMW through the use of osmotic distillation (OD). García-Castello et al. [24] measured average permeate flux values of about 0.7 kg/m^2^h when concentrating NF permeate by an OD process, in an operating time of 200 min, using a laboratory plant equipped with a Liqui-Cel^®^ Extra-Flow membrane module (Celgard LLC, Charlotte, NC, USA), containing microporous PP hollow fiber membranes and calcium chloride dehydrate (60% *w*/*w*), as stripping solution recirculated in the lumen side of the module. Similar average permeate flux values have been reported by Bazzarelli et al. [25] in the concentration of NF retentate by OD by using the same OD equipment and similar operating conditions.

### 2.2. Chemical Analyses

OMW phenolic compounds are well known for their remarkable antioxidant activity, which strongly suggests their re-utilization as nutraceuticals and additives in cosmetics and foodstuffs [26]. Herein, in order to evaluate the efficiency of the proposed concentration process, we selected (and quantified using ultra-high performance liquid chromatography, UHPLC) the most representative compounds identified in OMW, namely caffeic acid, *p*-coumaric acid, ferulic acid, 4-hydroxyphenyl acetate, hydroxytyrosol, oleuropein, tyrosol, vanillic acid, and verbascoside, as chemical markers.

In Table 1, the results related to the concentration of microfiltered OMW (feed) up to a WRF of 5.71, are reported. A concentration of all analyzed compounds is evident. Tyrosol, hydroxytyrosol, oleuropein, and verbascoside are the most abundant constituents. In particular, MD retentate has content of about five times greater of hydroxytyrosol and verbascoside, about six times of tyrosol, and about seven times greater of oleuropein, than the feed.

The sum of quantified phenolic compounds in the concentrated MD sample resulted in about 2770.7 mg/L. This value is 5.4-times greater than the phenolic content of the feed sample (513.1 mg/L); therefore, the concentration factor for phenolic compounds is in agreement with the final WRF of the MD process (5.71). On the other hand, El-Abbassi et al. [27] reported concentration factors for phenolic compounds of 1.72 and 1.4 after 9 h of MD with PTFE (TF200, Pall Gelman, Port Washington, NY, USA) and polyvinylidene fluoride (GVHP, Merck Millipore Inc., Billerica, MA, USA) flat-sheet hydrophobic membranes, respectively.

In the previous work, Tundis et al. [19] investigated a combination of pressure-driven membrane operations in a sequential design to produce a polyphenol-enriched fraction from the same OMW sample used in this study. OMW was microfiltered and submitted through NF and RO steps to concentrate phenolic compounds up to volume reduction factors of 4 and 2, respectively. The concentrated RO fraction presented a total content of phenolic compounds of 3071.1 mg/L, with hydroxytyrosol being the most abundant compound (1522.2 mg/L). Therefore, the results of this study confirm the possibility of achieving concentrated fractions with a similar content of phenolic compounds, but with a single concentration step (DCMD instead of NF/RO) operating at atmospheric pressure, with lower fouling, concentration polarization phenomena, and reduced energetic consumption. In addition, the feed temperature used in this study (40.1 °C) allows to avoid thermal degradation of phenolic compounds, as confirmed by the concentration factor and the final WRF of the process. Thermal degradation of phenolic compounds were reported by Kiai et al. [28] in the treatment of table olive wastewaters by DCMD with PTFE membranes supported by PP net (TF200, TF450, and TF1000, all from Gelman) at feed temperatures higher than 60 °C. Indeed, the decay in permeate flux observed for all selected membranes between 60 °C and 70 °C was attributed to the effect of the thermal degradation of phenolic compounds leading to by-products that could penetrate into the membrane pores. Galanakis et al. [29] reported that phenolic compounds of OMW are not destroyed by thermal treatment at 80 °C. Moreover, thermal treatment of OMW at 60 °C is known to reduce phenolic content due to the activation of endogenous polyphenol oxidases.

### 2.3. In Vitro Bioactivities

A perusal analysis of literature data revealed remarkable antioxidant and anti-obesity properties of phenolic compounds [4]. In this context, we analyzed, in comparison to the feed, the MD retentate that demonstrated to be a concentrated fraction of these phytochemicals, in particular of hydroxytyrosol, tyrosol, oleuropein, and verbascoside.

The potential hypoglycemic and hypolipidemic activity and the antioxidant effects are reported in Table 2 and Table 3. Moreover, α-amylase and α-glucosidase have been targeted as potential approaches for modulation of post-prandial hyperglycemia through the inhibition of the breakdown of complex carbohydrates to determine the reduction of meal-derived glucose absorption. Pancreatic lipase is a key enzyme regulating the absorption of triacylglycerols [30].

As reported in Table 2, both feed and MD retentate are able to inhibit α-amylase in a similar concentration-manner. Contrarily, α-glucosidase was more sensible to the action of MD retentate that showed an inhibitory concentration 50% (IC50) value of 63.4 μg/mL. The MD retentate was characterized by a higher content of hydroxytyrosol, tyrosol, oleuropein, and verbascoside with a promising α-amylase and α-glucosidase inhibitory activity. Indeed, Hadrich et al. [31] showed a strong α-glucosidase inhibitory activity (IC_50_ of 150 μM), higher than the positive control acarbose (IC_50_ of 200 μM). Oleuropein was less active with an IC50 of 400 μM. Interestingly, MD retentate inhibited lipase with an IC_50_ of 181.0 μg/mL in comparison to the feed that exhibited an IC_50_ of 400.8 μg/mL.

Four assays were applied to investigate the antioxidant potential of MD retentate, in comparison to the feed and some positive controls. As evident in Table 3, MD retentate was more active as antioxidant in all assays. Of particular interest is the result obtained in the DPPH test, with an IC_50_ of 9.8 μg/mL in comparison to the feed (IC_50_ of 97.2 μg/mL), and in the ABTS assay, with an IC_50_ of 0.4 μg/mL in comparison to the feed (IC_50_ of 1.2 μg/mL).

Moreover, in the β-carotene bleaching test, after 30 min of incubation, MD retentate exhibited an antioxidant activity of about eight times greater of the feed (IC_50_ of 3.5 μg/mL vs. 28.1 μg/mL, respectively).

We can suppose that the founded antioxidant activity was related to the high content of some phenolic compounds, including hydroxytyrosol and oleuropein.

Several previous studies have investigated the antioxidant activity of hydroxytyrosol [32,33]. The mechanism of action mainly includes the scavenging of free radicals and the inhibition of lipid peroxidation, and the prevention of metal ions production [34]. This is due to the presence of the catechol group in the structure of hydroxytyrosol and its capacity to act as a chelate of the intracellular iron. Strong radical scavenging activity of hydroxytyrosol towards DPPH, superior to that of vitamin E and BHT, was described by Visioli et al. [32]. Some cellular studies also reported the high antioxidant activity of this compound. In rat adrenal pheochromocytoma cells, pre-treatment with hydroxytyrosol at concentrations in the range 1–50 µM reduced the toxic effects induced by 6-hydroxydopamine and H_2_O_2_ [33]. In another work, performed in human hepatocarcinoma cells, with HepG2 oxidized by t-butyl hydroperoxide, the pre-treatment with hydroxytyrosol (10–40 µM) prevented cell damage and maintained the glutathione levels [35].

Hydroxytyrosol also exhibited antioxidant properties through its ability to stimulate the expression and the activity of key regulatory enzymes of oxidative stress, such as glutathione peroxidase, superoxide dismutase, and catalase [36,37]. The antioxidant-related health effects of hydroxytyrosol are supported by animal studies.

Granados-Principal et al. [38] demonstrated that the administration of hydroxytyrosol (0.5 mg/kg), 5 days/week for 6 weeks in Sprague–Dawley rats with breast cancer, reduced the production of reactive oxygen species (ROS) and restored, in mitochondria, the redox chain.

In another work, González-Santiago et al. [39] showed that hydroxytyrosol (4 mg/kg body weight, for one month) improved the antioxidant status and the blood lipid profile of the treated rabbits, and decreased the levels of total triglycerides and cholesterol.

Oleuropein demonstrated, in vitro, to inhibit copper sulfate-induced oxidation of low-density lipoproteins, assessed through a decrease in thiobarbituric acid-reacting substances and lipid peroxide by-product content [40,41]. The scavenging effects of oleuropein was demonstrated against hypochlorous acid, which is a potent oxidant species produced by neutrophil myeloperoxidase at the site of inflammation [32], as well as against nitric oxide [42]. Rabbits fed with a diet rich in oleuropein exhibited a higher serum antioxidant levels able to counteract low-density lipoprotein (LDL) oxidation, and a reduction of total, free, and esterified cholesterol levels, in comparison to rabbits that received a standard diet [43].

The whole results clearly confirm that DCMD can be an effective process for obtaining a phenolic-rich concentrate from OMW. In this view, this methodology could be a profitable process in relation to the exploitation of phenolic compounds. According to rough cost estimations, the price of crude polyphenols could reach up to 0.7 €/L [44].

Zaklis et al. [45] analyzed the sustainability of existing methods of OMW treatments, including physicochemical, biological, and advanced oxidation methods. The analyses showed that membrane filtration was one of the most effective processes in terms of organics reduction and economic viability, thanks to the profit derived from the exploitation of phenolic content and the fraction rich in nutrient components. The operational costs were calculated at around € 1,535,740 for the treatment of 50,000 tons of waste, which is equivalent to € 30.71 per treated m^3^ of OMW. The possible profit for the same amount of waste was calculated at € 1,875,000 for the phytotoxic fraction, corresponding to a profit of 42.5 €/m^3^ of treated OMW, and a net profit of 11.79 €/m^3^. Savarese et al. [46] also reported a combination of pressure-driven membrane operations and adsorbent resins for the separation and purification of phenolic compounds from OMW resulting from a three-phase milling system. Total unit costs of 125.01 €/m^3^ and 58.61 €/m^3^ were estimated for plants of capacity of 20 m^3^/day and 200 m^3^/day, respectively. The income coming from the selling of the antioxidant extracts was estimated of about 200 €/kg.

In agreement with other studies reported in literature [21,28], we believe that DCMD is also an efficient tool for high quality water production useful for irrigation or industrial uses, with resultants environmental benefits.

## 3. Materials and Methods

### 3.1. Chemicals and Reagents

Solvents of analytical grade were obtained from VWR International s.r.l. (Milan, Italy), solvents of HPLC grade were purchased from Carlo Erba Reagents (Milan, Italy). Acarbose was obtained from Serva (Heidelberg, Germany). Tween 20, ascorbic acid, Folin-Ciocalteu reagent, butylated hydroxytoluene (BHT), sulfuric acid, sodium carbonate, orlistat, 4-nitrophenyl octanoate (NPC), propyl gallate, 2,2-diphenyl-1-picrylhydrazyl (DPPH), β-carotene, tripyridyl triazine (TPTZ), 2,2′-azino-bis(3-ethylbenzothiazoline-6-sulfonic) acid (ABTS) solution, maltose, vanillic acid, linoleic acid, 4-nitrophenyl octanoate (NPC), α-amylase from porcine pancreas, porcine pancreatic lipase, α-glucosidase from *Saccharomyces cerevisiae*, *o*-dianisidine dihydrochloride, peroxidase/glucose oxidase (PGO), caffeic acid, hydroxytyrosol, verbascoside, tyrosol, 4-hydroxyphenyl acetate, *p*-coumaric acid, ferulic acid, and oleuropein were purchased from Sigma-Aldrich S.p.a. (Milan, Italy).

### 3.2. Feed Samples

OMW was kindly supplied by a three-phase olive mill unit located in the Calabria region (Olearia San Giorgio, Reggio Calabria, Italy). They were pre-treated in order to reduce fouling phenomena in MD testing and, consequently, to improve the performance of the MD membrane. In particular, raw wastewater was treated with sulfuric acid (95–98% purity) up to a final pH of 2.6, in order to achieve the coagulation and precipitation of suspended solids. Acidified OMW was prefiltered through a nylon filter, followed by a filtration with a wire mesh filter of 30–40 μm. This process allowed reducing the suspended solids content from 11.5% to 2.0%. Pre-treated OMW was submitted to a microfiltration process by using a pilot unit equipped with a multichannel ceramic (TiO_2_) membrane (Ceram Inside, from Tami Industries, nominal pore size of 0.1 μm, and membrane surface area of 0.35 m^2^), according to the procedure reported by Tundis et al. [19].

### 3.3. Membrane Distillation: Experimental Set-Up

**Figure 3:**
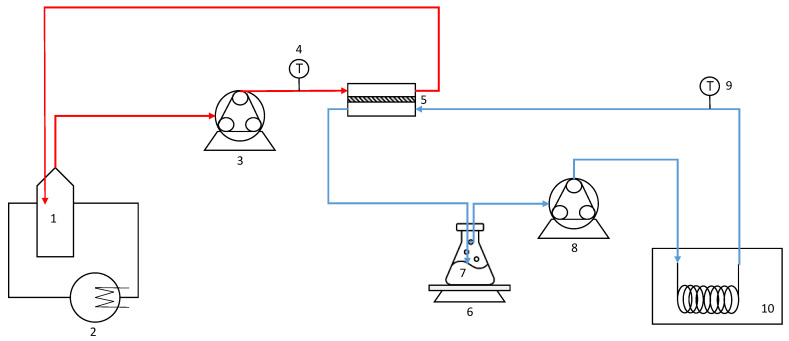
MD experimental set-up: 1, feed tank; 2, external heater; 3,8, peristaltic pump; 4,9, thermocouple; 5, membrane cell; 6, digital balance; 7, deionized water tank; 10, external cooling bath.

DCMD experiments were performed by using the experimental setup, schematically depicted in Figure 3. It was equipped with a membrane cell consisting of two compartments—the feed side and the permeate side. The module was positioned horizontally so that the feed solution flowed through the bottom compartment of the cell while the cooling water passed through the upper compartment. An external cooling bath was used for the regulation of the cold stream (permeate) temperature, while a thermostatic bath was used for the regulation of the feed stream (microfiltered OMW) temperature.

Feed and distilled water were contained in glass reservoirs and circulated counter-currently through the membrane cell at flowrates of 150 L/h and 80 L/h, respectively, using two peristaltic pumps. Two thermocouples (accuracy ± 0.1 °C) were used to measure the module inlet and outlet temperatures of both streams.

The membrane cell was equipped with a flat sheet polyvinylidene fluoride (PVDF) membrane, with a membrane pore size of 0.22 μm. The effective area of the membrane was 40 cm^2^. MD experiments were performed, maintaining the feed temperature (T_feed_) at 40.1 °C and the permeate temperature (T_p_) at 10.1 °C (ΔT = 30 °C).

The initial amount of microfiltered OMW of 300 g was treated up to achieve a concentrated sample of 84 g or 52.5 g, corresponding to a weight reduction factor (WRF) of 3.57 and 5.71, respectively. The amount of the extracted water was measured with a digital balance (Gibertini Elettronica, Milan, Italy) placed under the distilled water tank. The permeation flux (*J*) was calculated by using the following Equation (1):

(1) $$J=\frac{1}{A}\cdot \frac{dw}{dt}$$

where *w* is the collected permeate weight during a predetermined time (*t*) and *A* is the effective membrane area (4 × 10^−3^ m^2^).

Preliminary DCMD tests were carried out with distilled water as feed in order to evaluate the permeate flux achievable in absence of fouling phenomena. It was recirculated through the membrane cell at a flowrate (Q_f_) of 150 L/h while the permeate (distilled water) was recirculated at a flowrate (Q_p_) of 80 L/h. The temperature of the permeate side was maintained at 10.2 °C, while the hot side varied from 24.1 °C to 43.5 °C.

### 3.4. Ultra-High Performance Liquid Chromatography (UHPLC) Analyses

The identification and quantification of the most representative compounds identified in OMW was performed by using ultra-high performance liquid chromatography (UHPLC), as previously described [47]. An UHPLC PLATIN blue (Knauer, Germany), equipped with a binary pump system and a Knauer column C18 (100 × 2 mm, 1.8 μm), coupled with a Photo Diode Array Detector (PDA)-1 PLATIN blue (Knauer, Germany), was used. Clarity 6.2 (Clarity System Limited, Toronto, Canada) was used as the software. Feed and MD retentate were filtered by using a syringe filter of 0.22 μm. The mobile phase was a combination of two solutions: A (water acidified with acetic acid, pH 3.1) and B (acetonitrile). The following gradient of elution was used: 1) 95% A and 5% B, 0–3 min; 2) 95–60% A and 5–40% B, 3–15 min; and 3) 60–0% A and 40–100% B, 15–15.5 min. External standards, at concentrations in the range 1–100 mg/L, such as caffeic acid, verbascoside, *p*-coumaric acid, ferulic acid, 4-hydroxyphenyl acetate, hydroxytyrosol, oleuropein, vanillic acid, and tyrosol, were used for the quantification.

Regression coefficient, limit of detection, and limit of quantification were reported as mean of obtained values. The single values for detected phenolic compounds are reported in Table 4.

### 3.5. Enzymes Inhibitory Activities

#### 3.5.1. α-Amylase

This test was done following the procedure previously reported [19]. In brief, the α-amylase solution was prepared by dissolving the enzyme (25.3 mg) in cold distilled water (100 mL) [40,41]. The addition of a 3,5-dinitrosalicylic acid solution to a sodium potassium tartrate solution allowed to prepare the colorimetric reagent. Samples (concentrations in the range 12.50–1000 μg/mL) were added to the starch solution and left to react with the enzyme at room temperature. The absorbance was measured at 540 nm and acarbose was used as a positive control.

#### 3.5.2. α-Glucosidase

For this assay, the method previously described was followed [19]. Briefly, samples (concentrations in the range 12.50–1000 μg/mL) were stirred into a maltose solution at 37 °C. The reaction, started by adding the enzyme, was stopped by adding a perchloric acid solution after 30 min of incubation at 37 °C. The supernatant of tube of the first step was mixed with *o*-dianisidine color reagent (DIAN) solution and peroxidase-glucose oxidase (PGO) system-color reagent solution, and was left to incubate at 37 °C for 30 min. The absorbance was measured at 540 nm. Acarbose was used as a positive control

#### 3.5.3. Lipase

Pancreatic lipase inhibitory activity was determined as previously reported by El-shiekh et al. [48]. A solution 5 mM of 4-nitrophenyl octanoate (NPC) in dimethyl sulfoxide, an aqueous solution of lipase (1 mg/mL), and Tris-HCl buffer (pH 8.5) were prepared. The mixture—consisting of the enzyme, NPC, samples (at concentrations in the range 2.5–40.0 mg/mL), and buffer—was incubated at 37 °C, for 30 min. The absorbance was measured at 405 nm. Orlistat was used as a positive control.

### 3.6. Antioxidant Activity

The in vitro antioxidant effects of our samples was analyzed by using the following tests: β-carotene bleaching test; 2,2′-azino-bis(3-ethylbenzothiazoline-6-sulphonic) acid (ABTS) diammonium salts test; 2,2-diphenyl-1-picrylhydrazyl (DPPH); and ferric reducing activity power (FRAP).

#### 3.6.1. β-Carotene Bleaching Test

The β-carotene bleaching test was done following the method previously described [49]. A solution of β-carotene, linoleic acid, and Tween 20, was prepared and added to a 96-well microplate containing samples at concentrations ranging from 2.5 to 100 μg/mL. The microplate was located at 45 °C. The absorbance was read at 470 nm against a blank at *t* = 0, after 30 min of incubation, and after 60 min of incubation. Propyl gallate was chosen as a positive control.

#### 3.6.2. ABTS Test

The radical scavenging ability of our samples were investigated by the ABTS test [49]. In brief, a solution of ABTS radical cation and potassium persulfate was prepared and stored at 25 °C for 12 h. Then, this solution was diluted with ethanol, read to obtain an absorbance of 0.70 at 734 nm, and added to samples at concentrations ranging from 1 to 400 μg/mL. After 6 min, the absorbance was read at 734 nm. Ascorbic acid was used as positive control.

#### 3.6.3. DPPH Test

In this assay, a ethanolic solution of DPPH 0.25 mM and samples at different concentrations in the range 1–1000 μg/mL were prepared, shaken, and incubated at room temperature for 30 min [49]. The positive control was ascorbic acid. The bleaching of DPPH was determined by measuring the absorbance at 517 nm.

#### 3.6.4. FRAP Test

This assay is based on the reduction of Fe^3+^ tripyridyl triazine (TPTZ) complex (colorless) to Fe^2+^-TPTZ (blue colored) formed at low pH. Antioxidants that react in this test are those that can reduce the Fe^3+^-TPTZ salt to its colored Fe^2+^-TPTZ form. The absorbance was measured at 595 nm after 30 min of incubation at room temperature. Samples were tested at the concentration of 2.5 mg/mL and the positive control was butylated hydroxytoluene (BHT) [49].

### 3.7. Statistical Analysis

IC_50_ (the concentration giving 50% inhibition) values were calculated by using Prism GraphPad Prism version 4.0 for Windows (GraphPad Software, San Diego, CA, USA). Data are reported as means ± standard deviation. Differences within and between groups were assessed by ANOVA (one-way analysis of variance test) and multi-comparison Dunnett’s test (α = 0.05).

## 4. Conclusions

The results obtained in the present study confirm that direct contact membrane distillation (DCMD) can be applied successfully for phenolic concentration of olive mill wastewater (OMW).

A concentrated fraction containing 2770.7 mg/L of phenolic compounds (with hydroxytyrosol the most abundant compound, 1902.9 mg/L) was obtained from microfiltered OMW at feed and permeate temperatures of 40.1 and 10.1 °C, respectively. The concentration factor for phenolic compounds was in agreement with the weight reduction factor of the process, indicating no thermal degradation of bioactive compounds. Additional advantages concern the possibility of producing concentrated phenolic fractions in a single concentration step, operating at lower transmembrane pressures in comparison to pressure-driven membrane systems, with lower fouling and concentration polarization phenomena and reduced energetic consumption.

Overall, the MD retentate proves to be a more efficient scavenger of free radicals than the feed and, in some cases, of the positive control in applied antioxidant tests. Moreover, MD retentate exhibited promising hypoglycemic and hypolipidemic properties. In particular, α-glucosidase activity was inhibited more in the presence of the MD retentate compared to the feed. This difference may be related to the high content in the retentate of some phenolics—such as hydroxytyrosol and oleuropein—that demonstrated to possess promising hypoglycemic activity. In conclusion, this study adds more value to OMW for further studies as a potential source of lead bioactive compounds to prevent and/or to treat type 2 diabetes and obesity.

By referring to the practical applications, we believe that, despite benefits arising from exploitation of phenolic compounds and production of high quality water production, with resulting environmental benefits, the main limitation for OMW treatment by DCMD is still represented by the relatively low permeate fluxes. Further investigations focused on the use of new membrane materials, the optimization of operating conditions, and long-term experiments are needed to estimate the real application of this technology.

## Figures and Tables

**Table 1:** Analysis via ultra-high performance liquid chromatography (UHPLC) of phenolic compounds in feed and MD retentate samples. Data are expressed as the means ± relative standard deviation (RSD) of three independent observations.

Compound	Feed	MD Retentate
	(mg/L)	(mg/L)
Caffeic acid	6.0 ± 0.3	25.9 ± 1.2
*p*-Coumaric acid	0.9 ± 0.01	5.4 ± 0.4
Ferulic acid	3.8 ± 0.4	21.2 ± 0.5
4-Hydroxyphenlyl acetate	8.5 ± 0.9	44.9 ± 2.5
Hydroxytyrosol	367.7 ± 3.6	1902.9 ± 7.6
Oleuropein	35.2 ± 2.4	235.2 ± 2.0
Tyrosol	41.0 ± 2.0	257.9 ± 3.5
Vanillic acid	1.0 ± 0.3	5.5 ± 0.4
Verbascoside	49.0 ± 1.8	271.8 ± 2.5

**Table 2:** Hypoglycemic and hypolipidemic activity (inhibitory concentration 50% (IC_50_) value (μg/mL)).

Sample	α-Amylase	α-Glucosidase	Lipase
Feed	135.5 ± 10.2 ****	83.1 ± 8.2 ****	400.8 ± 45.2 ****
MD retentate	125.0 ± 10.2 ****	63.4 ± 2.6 ***	181.0 ± 23.4 ****
Positive control			
Acarbose	50.0 ± 1.4	35.5 ± 1.1	
Orlistat			37.5 ± 1.2

MD, membrane distillation. Data are expressed as means ± S.D. (*n* = 3). Differences within and between groups were evaluated by one-way ANOVA followed by a multi-comparison Dunnett’s test (α = 0.05): **** *p* < 0.0001, *** *p* < 0.001, compared with the positive control.

**Table 3:** In vitro antioxidant effects.

Sample	ABTS TestIC_50_ (μg/mL)	DPPH Test IC_50_ (μg/mL)	FRAP Test IC_50_ (μM Fe (II)/g)	β-Carotene Bleaching Test IC_50_ (μg/mL)	
				30 min	60 min
Feed	1.2 ± 0.20 *	97.2 ± 5.2 ****	67.7 ± 4.2 ^ns^	28.1 ± 2.8 ****	41.7 ± 2.3 ****
MD retentate	0.4 ± 0.03 ***	9.8 ± 0.9 ^ns^	101.2 ± 8.0 ****	3.5 ± 1.5	25.4 ± 2.6 ****
Positive control					
Ascorbic acid	1.7 ± 0.2	5.0 ± 0.8			
BHT			63.2 ± 4.3		
Propyl gallate				1.0 ± 0.01	1.0 ± 0.01

MD, membrane distillation. Data are expressed as means ± S.D. (*n*= 3). Differences within and between groups were evaluated by one-way ANOVA followed by a multi-comparison Dunnett’s test (α = 0.05): **** *p* < 0.0001, *** *p* < 0.001, * *p* < 0.1 compared with the positive control; ns: not significant.

**Table 4:** Regression coefficient (r^2^), limit of detection (LOD), and limit of quantification (LOQ) of detected phenolic compounds.

	Regression Equation	r^2^	LOD (mg/L)	LOQ (mg/L)
Caffeic acid	*y* = 111.97*x* − 84.67	0.9997	0.078	2.52
p-Coumaric acid	*y* = 114.17*x* + 35.47	0.9999	0.081	4.01
Ferulic acid	*y* = 127.18*x* − 72.81	0.9998	0.077	4.33
4-Hydroxyphenlyl acetate	*y* = 26.28*x* − 27.44	0.9996	0.075	2.32
Hydroxytyrosol	*y* = 25.77*x* − 24.41	0.9999	0.088	4.51
Oleuropein	*y* = 46.63*x* + 51.21	0.9997	0.08	4.15
Tyrosol	*y* = 41.25*x* − 49.09	0.9998	0.081	2.31
Vanillic acid	*y* = 79.88*x* + 18.07	0.9997	0.079	2.22
Verbascoside	*y* = 64.22*x* − 557.92	0.9996	0.08	3.33

## Data Availability

Not applicable.

## Sample Availability

Samples of the compounds are not available from the authors.

## References

[1] Rahmanian N., Jafari S.M., Galanakis C.M. (2014). Recovery and removal of phenolic compounds from olive mill wastewater. J. Am. Oil Chem. Soc..

[2] Roig A., Cayuela M.L., Sánchez-Monedero M.A. (2006). An overview on olive mill wastes and their valorisation methods. Waste Manag..

[3] Bulotta S., Celano M., Lepore S.M., Montalcini T., Pujia A., Russo D. (2014). Beneficial effects of the olive oil phenolic components oleuropein and hydroxytyrosol: Focus on protection against cardiovascular and metabolic diseases. J. Transl. Med..

[4] Cicerale S., Lucas L.J., Keast R.S.J. (2012). Antimicrobial, antioxidant and anti-inflammatory phenolic activities in extra virgin olive oil. Curr. Opin. Biotechnol..

[5] Farooqi A.A., Fayyaz S., Sanches Silva A., Sureda A., Nabavi S.F., Mocan A., Nabavi S.F., Mocan A., Nabavi S.M., Bishayee A. (2017). Oleuropein and cancer chemoprevention: The link is hot. Molecules.

[6] Hassen I., Casabianca H., Hosni K. (2015). Biological activities of the natural antioxidant oleuropein: Exceeding the expectation—A mini-review. J. Funct. Food..

[7] El-Abbassi A., Kiai H., Hafidi A. (2012). Phenolic profile and antioxidant activities of olive mill wastewater. Food Chem..

[8] Didaskalou C., Buyuktiryaki S., Kecili R.S., Fonte C.P., Szekely G. (2017). Valorisation of agricultural waste with an adsorption/nanofiltration hybrid process: From materials to sustainable process design. Green Chem..

[9] Cassano A., Conidi C., Giorno L., Drioli E. (2013). Fractionation of olive mill wastewaters by membrane separation techniques. J. Hazard. Mater..

[10] Ochando-Pulido M.J., Martinez-Ferez A. (2015). On the recent use of membrane technology for olive mill wastewater purification. Membranes.

[11] Alkhudhiri A., Darwish N., Hilal N. (2012). Membrane distillation: A comprehensive review. Desalination.

[12] Bouchrit R., Boubakri A., Hafiane A., Bouguecha A.-T.S. (2015). Direct contact membrane distillation: Capability to treat hyper-saline solution. Desalination.

[13] Liu H., Wang J. (2013). Treatment of radioactive wastewater using direct contact membrane distillation. J. Hazard. Mater..

[14] Criscuoli A., Zhong J., Figoli A., Carnevale M.C., Huang R., Drioli E. (2008). Treatment of dye solutions by vacuum membrane distillation. Water Res..

[15] Conidi C., Castro-Muñoz R., Cassano A. (2020). Membrane-based operations in the fruit juice processing industry: A review. Beverages.

[16] Quist-Jensen C.A., Macedonio F., Conidi C., Cassano A., Aljlil S., Alharbi O.A., Drioli E.E. (2016). Direct contact membrane distillation for the concentration of clarified orange juice. J. Food Eng..

[17] El-Bourawi M.S., Ding Z., Ma R., Khayet M. (2006). A framework for better understanding membrane distillation separation process. J. Membr. Sci..

[18] Susanto H. (2011). Towards practical implementations of membrane distillation. Chem. Eng. Process..

[19] Tundis R., Conidi C., Loizzo M.R., Sicari V., Cassano A. (2020). Olive mill wastewater polyphenol-enriched fractions by integrated membrane process: A promising source of antioxidant, hypolipidemic and hypoglycaemic compounds. Antioxidants.

[20] El-Abbassi A., Kiai H., Hafidi A., Garcia-Payo M.C., Khayet M. (2012). Treatment of olive mill wastewater by membrane distillation using polytetrafluoroethylene membranes. Sep. Purif. Technol..

[21] El-Abbassi A., Hafidi A., Khayet M., García-Payo M.C. (2013). Integrated direct contact membrane distillation for olive mill wastewater treatment. Desalination.

[22] Carnevale M.C., Gnisci E., Hilal J., Criscuoli A. (2016). Direct contact and vacuum membrane distillation application for the olive mill wastewater treatment. Sep. Purif. Technol..

[23] Gryta M. (2008). Fouling in direct contact membrane distillation process. J. Membr. Sci..

[24] García-Castello E., Cassano A., Criscuoli A., Conidi C., Drioli E. (2010). Recovery and concentration of polyphenols from olive mill wastewaters by integrated membrane system. Water Res..

[25] Bazzarelli F., Piacentini E., Poerio T., Mazzei R., Cassano A., Giorno L. (2016). Advances in membrane operations for water purification and biophenols recovery/valorization from OMWWs. J. Membr. Sci..

[26] Galanakis C.M. (2012). Recovery of high added-value components from food wastes: Conventional, emerging technologies and commercialized applications. Trends Food Sci. Technol..

[27] El-Abbassi A., Hafidi A., García-Payo M.C., Khayet M. (2009). Concentration of olive mill wastewater by membrane distillation for polyphenols recovery. Desalination.

[28] Kiai H., Garcia-Payo M.C., Hafidi A., Khayet M. (2014). Application of membrane distillation technology in the treatment of table olive wastewaters for phenolic compounds concentration and high quality water production. Chem. Eng. Process..

[29] Galanakis C.M., Tornberg E., Gekas V. (2010). The effect of heat processing on the functional properties of pectin contained in olive mill wastewater. LWT Food Sci. Technol..

[30] Adriano C.d.C., Silva A.P.d.S., Soares J.C., de Alencar S.M., Handa C.L., Cordeiro K.S., Figueira M.S., Sampaio G.R., Torres E.A.F.S., Shahidi F. (2021). Do flavonoids from durum wheat contribute to its bioactive properties? A prospective study. Molecules.

[31] Hadrich F., Bouallagui Z., Junkyu H., Isosa H., Sayadi S. (2015). The α-glucosidase and α-amylase enzyme inhibitory activity of hydroxytyrosol and oleuropein. J. Oleo Sci..

[32] Visioli F., Bellomo G., Galli C. (1998). Free radical-scavenging properties of olive oil polyphenols. Biochem. Biophys. Res. Commun..

[33] Peng S., Zhang B., Yao J., Duan D., Fang J. (2015). Dual protection of hydroxytyrosol, an olive oil polyphenol, against oxidative damage in PC12 cells. Food Funct..

[34] Robles-Almazan M., Pulido-Moran M., Moreno-Fernandez J., Ramirez-Tortosa C., Rodriguez-Garcia C., Quiles J.L., Ramirez-Tortosa M.C. (2018). Hydroxytyrosol: Bioavailability, toxicity, and clinical applications. Food Res. Int..

[35] Goya L., Mateos R., Bravo L. (2007). Effect of the olive oil phenol hydroxytyrosol on human hepatoma HepG2 cells. Eur. J. Nutr..

[36] Vlavcheski F., Young M., Tsiani E. (2019). Antidiabetic effects of hydroxytyrosol: In vitro and in vivo evidence. Antioxidants.

[37] Zhu L., Liu Z., Feng Z., Hao J., Shen W., Li X., Sun L., Sharman E., Wang Y., Wertz K. (2010). Hydroxytyrosol protects against oxidative damage by simultaneous activation of mitochondrial biogenesis and phase II detoxifying enzyme systems in retinal pigment epithelial cells. J. Nutr. Biochem..

[38] Granados-Principal S., El-azem N., Pamplona R., Ramirez-Tortosa C., Pulido-Moran M., Vera-Ramirez L., Quiles J.L., Sanchez-Rovira P., Naudí A., Portero-Otin M. (2014). Hydroxytyrosol ameliorates oxidative stress and mitochondrial dysfunction in doxorubicin-induced cardiotoxicity in rats with breast cancer. Biochem. Pharmacol..

[39] González-Santiago M., Martín-Bautista E., Carrero J.J., Fonollá J., Baró L., Bartolomé M.V., Gil-Loyzaga P., López-Huertas E. (2006). One-month administration of hydroxytyrosol, a phenolic antioxidant present in olive oil, to hyperlipemic rabbits improves blood lipid profile, antioxidant status and reduces atherosclerosis development. Atherosclerosis.

[40] Visioli F., Bellomo G., Montedoro G., Galli C. (1995). Low density lipoprotein oxidation is inhibited in vitro by olive oil constituents. Atherosclerosis.

[41] Visioli F., Galli C. (1994). Oleuropein protects low density lipoprotein from oxidation. Life Sci..

[42] De la Puerta R., Dominguez M.E.M., Ruiz-Gutierrez V., Flavill J.A., Hoult J.R.S. (2001). Effects of virgin olive oil phenolics on scavenging of reactive nitrogen species and upon nitrergic neurotransmission. Life Sci..

[43] Coni E., Di Benedetto R., Di Pasquale M., Masella R., Modesti D., Mattei R., Carlini E.A. (2000). Protective effect of oleuropein, an olive oil biophenol, on low density lipoprotein oxidizability in rabbits. Lipids.

[44] Paraskeva C.A., Papadakis V.G., Kanellopoulou D.G., Koutsoukos P.G., Angelopoulos K.C. (2007). Membrane filtration of olive mill wastewater and exploitation of its fractions. Water Environ. Res..

[45] Zagklis D.P., Arvaniti E.C., Papadakis V.G., Paraskeva C.A. (2013). Sustainability analysis and benchmarking of olive mill wastewater treatment methods. J. Chem. Technol. Biotechnol..

[46] Savarese M., De Marco E., Falco S., D’Antuoni I., Sacchi R. (2016). Biophenol extracts from olive oil mill wastewaters by membrane separation and adsorption resin. Int. J. Food Sci. Technol..

[47] Romeo R., De Bruno A., Imeneo V., Piscopo A., Poiana M. (2020). Impact of stability of enriched oil with phenolic extract from olive mill wastewaters. Foods.

[48] El-shiekh R.A., Al-Mahdy D.A., Hifnawy M.S., Abdel-Sattar E.A., El-Shiekh R.A., Al-Mahdy D.A., Hifnawy M.S., Abdel-Sattar E.A. (2019). In-vitro screening of selected traditional medicinal plants for their anti-obesity and antioxidant activities. S. Afr. J. Bot..

[49] Leporini M., Bonesi M., Loizzo M.R., Passalacqua N.G., Tundis R. (2020). The essential oil of *Salvia rosmarinus* Spenn. From Italy as a source of health-promoting compounds: Chemical profile and antioxidant and cholinesterase inhibitory activity. Plants.

